# The validity and applications of the analgesia nociception index: a narrative review

**DOI:** 10.3389/fsurg.2023.1234246

**Published:** 2023-08-10

**Authors:** Bill Hum, Alexa Christophides, Zhaosheng Jin, Murad Elias, Kamil Taneja, Sergio D. Bergese

**Affiliations:** ^1^ Department of Anesthesiology, Stony Brook University Health Science Center, Stony Brook, NY, United States; ^2^ Medical Scientist Training Program, Renaissance School of Medicine, Stony Brook University, Stony Brook, NY, United States

**Keywords:** analgesia, nociception, analgesia nociception index, monitoring, pain

## Abstract

Pain refers to the subjective, unpleasant experience that is related to illness or injury. In contrast to pain, nociception refers to the physiological neural processing of noxious stimuli, such as intra-operative surgical stimuli. One novel device, the Analgesia Nociception Index (ANI), aims to objectively measure intra-operative nociception by analyzing the heart rate variability in patients undergoing surgery. Through this method of nociceptive monitoring, the ANI device aims to provide an objective, continuous evaluation of patient comfort levels and allow anesthesiologists to better manage surgical stress and patient analgesia, perhaps with even better efficacy than current practices used to assess nociception. Additionally, ANI may have clinical application in settings outside of the operating room, such as in the intensive care unit. In this narrative review, we compiled and summarized the findings of many studies that have investigated ANI's validity and applications in different clinical settings. Currently, the literature appears mostly supportive of ANI's ability to detect nociception in both surgical and non-surgical settings. However, the ability for ANI to provide clinical benefits, such as decreased intra-operative opioid use, post-operative opioid use, and post-operative pain compared to standard practices appear controversial. Because of the wide variety of methodology, clinical settings, patient populations, and limitations in these studies, more investigation of ANI is needed before any firm conclusions can be drawn on its clinical benefits.

## 1. Introduction

Nociception is the body's neural process of encoding noxious stimuli. Numerous methods were developed to help clinicians assess nociception, such as monitoring hemodynamic parameters. Assessing changes in hemodynamic parameters can be especially useful intra-operatively, as it allows anesthesiologists to monitor their patients’ nociceptive response to surgical stimuli, optimize opioid administration, reduce pain, and improve post-operative outcomes.

Parameters such as blood pressure (BP) and heart rate (HR) may provide insight in a patient's nociception, but may be subject to confounding factors and not always accurately reflect nociception. To address this, many new devices have been developed to monitor nociception more accurately, such as the Analgesia Nociception Index (ANI) developed by MDoloris. ANI obtains electrocardiogram (ECG) data through two electrodes placed on the patient's chest and analyzes the interval between the R-R waves of the ECG. The changes in the R-R intervals, also known as the heart rate variability (HRV), are influenced by changes in sympathetic and parasympathetic tone (1). Changes in HRV in the high frequency (HF) range (0.15–0.40 Hz) are influenced by parasympathetic activity, whereas changes in HRV in the low frequency (LF) range (0.04–0.15 Hz) are influenced by both sympathetic and parasympathetic activities (2). A decrease in HF HRV or an increase in LF HRV suggests a decrease in parasympathetic tone and subsequently was shown to be indicative of pain or unpleasant stimuli (3, 4). With this information, ANI creates a value from 0 to 100, where a value greater than 50 indicates adequate analgesia (high parasympathetic tone) and a value less than 50 indicates nociception (a high sympathetic tone) and therefore inadequate analgesia and a likely chance of a hemodynamic response occurring within a few minutes.

Given ANI's potential in managing anesthetized patients and ease of use, many studies assessed for its clinical benefits in the intra-operative setting, the post-anesthesia care unit (PACU), and the intensive care unit (ICU). Additionally, many studies also assessed ANI's clinical relevance in various patient populations, such as anesthetized, pediatric, critically ill, maternal, and septic patients. PubMed search terms utilized for study selection included “nociception,” “analgesia,” “analgesia nociception index,” “ANI,” and “pain monitoring.” Following the search, two co-authors chose studies independently for inclusion by considering their study design, clinical importance, and journal characteristics. In this narrative review, we summarized the selected studies’ results on ANI's validity and other applications.

## 2. Validation of ANI's ability to detect intra-operative nociception and predict hemodynamic responses

### 2.1. Validation of ANI using artificial noxious stimuli

To validate ANI's ability to detect nociceptive surgical stimuli, four studies used an artificial noxious stimulus and monitored fluctuations in ANI values and hemodynamic responses. In theory, if a change in ANI preceded a hemodynamic response (such as a significant increase in BP or HR), this would support ANI's ability to predict whether a hemodynamic response will occur and provide anesthesiologists better insight in managing anesthetized patients.

In Gruenewald et al. and Susano et al., tetanic noxious stimulation was applied to patients with simultaneous monitoring of their ANI values (5, 6). Both studies had similar findings: ANI significantly decreased after the introduction of a noxious stimulus (i.e., ANI reflected nociception); however, there were no significant changes in hemodynamic responses (HR and BP). In Susano et al., one possible suggestion for the lack of change in hemodynamics was that ANI could be better than traditional hemodynamics at reflecting noxious stimulation (6). Alternatively, it is possible that noxious stimulus applied in the study was simply not strong enough to lead to changes in hemodynamic parameters.

Funcke et al. had both a similar and different conclusion compared to the previous two studies: ANI's prediction probability (P_K_) for detecting tetanic noxious stimuli was 0.98 (where P_K_ values range from 0.5 to 1; 1 representing a perfect prediction and 0.5 representing mere chance), with a sensitivity and specificity of 87.9% and 98.5% respectively (7). Contrary to the previous two studies, Funcke et al. found that ANI's prediction probability for a hemodynamic response (defined in their study as “increase in heart rate or blood pressure by >5 beats/min or >5 mmHg, respectively, or >10%”) was 0.70 with a sensitivity and specificity of 20.6% and 46.8%, respectively. Funcke et al. concluded as ANI having limited predictive value for hemodynamic responses.

Jozefowicz et al. had a contradictory finding compared to the previous three studies (8). In patients who received a tetanic noxious stimulation prior to tracheal intubation, there was no significant difference in ANI between patients who had a hemodynamic response and those who did not. Additionally, during intubation, the ability for ANI to predict a hemodynamic response was found to not be reliable given that the area under a receiver operating characteristic curve (AUC ROC) was 0.61 (Table 1).

**Table 1:** Summary of ANI's validity in detecting experimental and intra-operative nociception and predicting hemodynamic responses.

Study	ANI reflects nociception?	ANI has hemodynamic predictability?	Noxious stimuli	Patient age group	Sample size
Gruenewald et al. (5)	Yes	No	Artificial Tetanic Wrist Stimulation	Adults	25
Susano et al. (6)	Yes	No	Artificial Tetanic Electrical Stimulation	Adults	16
Funcke et al. (7)	Yes	Limited	Artificial Tetanic Electrical Stimulation	Adults	38
Jozefowicz et al. (8)	No	No	Artificial Tetanic Electrical Stimulation and Intubation	Adults	13
Ledowski et al. (9)	Yes	Yes	Varied Surgical Procedures	Adults	30
Jeanne et al. (2012) (10)	Yes	Yes	Total Knee Replacement	Adults	27
Jeanne et al. (2014) (11)	Yes	Yes	Laparoscopic Abdominal Surgery	Adults	15
Boselli et al. (2015) (12)	Yes	Yes	Suspension Laryngoscopy	Adults	50
Boselli et al. (2016) (13)	Yes	Yes	Ear-Nose-Throat or Orthopedic Surgery	Adults	128
Sriganesh et al. (14)	Yes	Not assessed	Laryngoscopy/Tracheal Intubation during Neurosurgery	Adults	60
Kommula et al. (15)	Yes	Not assessed	Craniotomy	Adults	21
Anderson et al. (16)	Yes	Not assessed	Laparoscopic Cholecystectomy	Adults	65
Xie et al. (17)	Yes	Not assessed	Abortion	Adults	98
Migeon et al. (18)	Yes	Not assessed	Skin Incision	Pediatric	58
Avez-Couturier et al. (19)	Yes	Not assessed	Muscle Biopsy	Pediatric	26
Weber et al. (20)	Yes	Not assessed	Various Surgeries	Pediatric	131
Julien-Marsollier et al. (21)	Yes	Not assessed	Skin Incision	Pediatric	49

### 2.2. Validation of ANI in clinical settings with adult patients

The following studies assessed both ANI's ability to detect nociceptive surgical and predict hemodynamic responses in various clinical settings. In these five studies with different noxious stimuli, the conclusions were similar: ANI significant decreased after a nociceptive stimulus and was followed by a significant change in hemodynamic parameters (defined as a minimum of 10% increase in BP or HR in Ledowski et al. and a minimum of 20% increase in BP or HR in Jeanne et al. and Boselli et al.) (9–13). As such, each study concluded that ANI reflected nociception and could predict hemodynamic changes following nociceptive stimuli. More notably, Ledowski et al. also demonstrated a significant increase in ANI after fentanyl administration, indicating adequate analgesia and decreased nociception after analgesic use (9) (Table 1).

### 2.3. Validation of ANI in clinical settings with pediatric patients

Many studies have also evaluated the validity of ANI to detect nociception in pediatric patients as well. In fact, four different studies all found evidence that supports ANI's ability to detect nociception in pediatric patients (18–21). These studies had similar methodology: measuring and comparing ANI values before and after noxious surgical stimuli. In addition to these findings, two studies also suggested that ANI may be better predictors of nociception than hemodynamic parameters. In Weber et al., when ANI was <50 (indicative of nociception) at the time of analgesic administration, ANI values would increase above 60 within 2 min, whereas there was no observed change in HR (20). From this observation, Weber et al. suggested that ANI may be better than HR at predicting nociception. Julien-Marsollier et al.'s AUROC analysis (predictive value) for ANI to detect nociceptive surgical stimuli was >0.75, whereas the AUROC values for HR, systolic BP, diastolic BP, and mean arterial pressure were 0.51, 0.60, 0.57, and 0.58 respectively (21). These authors concluded that hemodynamic parameters had little predictive value for noxious surgical stimuli compared to ANI (21) (Table 1).

### 2.4. ANI and intra-operative opioid use

Another potential application of ANI is optimizing intra-operative opioid consumption. Many studies have assessed this potential to determine if ANI-guided opioid administration alongside standard practice would lead to decreased intra-operative opioid use compared to standard practice. Three studies had a similar methodology: comparing the intra-operative opioid use in an ANI-guided group and a standard practice (control) group during surgery. Of the three studies, Soral et al. and Dundar et al. assessed remifentanil consumption, whereas Gall et al. assessed sufentanil consumption (22–24). All three studies, which involved three different types of surgeries, found a significantly lower opioid use in patients who underwent surgery with ANI monitoring compared to patients who were assessed via conventional means (Table 2).

**Table 2:** Summary of intra-operative opioid sparing with ANI.

Study	Decrease in intra-operative opioids?	Opioid used	Noxious stimulation	Sample size
Soral et al. (22)	Yes	Remifentanil	Colonoscopy	102
Dundar et al. (23)	Yes	Remifentanil	Breast Surgeries	44
Gall et al. (2017) (24)	Yes	Sufentanil	Bariatric Surgeries	60
Dostalova et al. (2019) (25)	No	Sufentanil	Neurosurgical Spinal Procedures	72
Tribuddharat et al. (2021) (26)	No	Fentanyl	Elective Mastectomy	60
Szental et al. (2015) (27)	No	Fentanyl	Laparoscopic Cholecystectomy	120

While those studies found that ANI guidance decreased intra-operative opioid consumption, some studies did not find a significant difference in opioid use between ANI-guided protocols and conventional methods (25–27). However, one notable conclusion mentioned by Dostolova et al. was that while ANI use did not lead to intra-operative opioid sparing, it still has potential clinical utility as there was also no significant difference in post-operative cortisol levels, pain scores, or complication rates (25) (Table 2).

## 3. ANI in the post-operative setting

### 3.1. Validation of ANI in detecting post-operative nociception

In addition to minimizing intra-operative opioid consumption, many studies were conducted on whether nociception could be detected reliably post-operatively. In these studies, ANI values were measured and compared post-operatively with a subjective pain assessment scale, such as the Numeric Rating Scale (NRS) or the Face, Legs, Activity, Cry, Consolability scale for pediatric patients (FLACC). In Boselli et al., there was a significantly negative linear relationship between the NRS and ANI values, with a sensitivity and specificity for ANI values <57 to detect an NRS >3 was 78% and 80% respectively, with an AUC ROC of 0.86 within 10 min of PACU arrival (28). Additionally, the sensitivity and specificity for ANI <48 to detect an NRS >7 was 92% and 82% respectively, with an AUC ROC of 0.91. Abdullayev et al. also found a significantly negative linear relationship between ANI and NRS scores (*r*^2^ = −0.312, *p* = 0.001) (29). Both studies' findings are consistent with ANI's intended clinical use: a higher ANI value (indicative of less nociception/sympathetic tone) should inversely correlate with a lower self-reported pain score. Similarly, Gall et al. (2015) findings support ANI's ability to predict post-operative pain in pediatric patients: children who had a surgical procedure had significantly lower ANI values (indicative of nociception) upon arrival to the PACU compared to children who underwent imaging; ANI and FLACC values were found to have a significantly negative linear relationship as well (30). Lastly, while Logier et al. did not compare post-operative ANI values to a pain scale, they found that post-operative ANI values significantly increased (indicative of less nociception) upon administration of truncal analgesia (31) (Table 3).

**Table 3:** Summary of ANI's validity in detecting post-operative nociception.

Study	ANI reflects post-operative nociception?	Pain assessment	ANI correlation with pain assessment	Sample size
Boselli et al. (2013) (28)	Yes	NRS	Negative Linear Relationship	200
Abdullayev et al. (29)	Yes	NRS	Negative Linear Relationship	107
Gall et al. (2015) (30)	Yes	FLACC	Negative Linear Relationship	62
Logier et al. (31)	Yes	N/A	N/A	9
Parker et al. (2013) (32)	No	NRS	“Small, but statistically significant negative correlations”	120

One study by Parker et al., however, concluded that ANI was not able to reflect post-operative pain as measured by the NRS scale (32). This study also compared NRS and ANI values upon arrival to the PACU. While there was a statistically significant negative correlation between NRS and ANI, the correlation was not strong (spearman's ro coefficient = −0.075) and that ANI's ability to distinguish NRS scores of 0 from NRS scores of 6–10 had low sensitivity and specificity (Table 3).

### 3.2. Intra-operative ANI use and post-operative pain

Given the studies validating the use of ANI to assess post-operative nociception, the next steps in exploring the use of ANI would be to evaluate whether intra-operative ANI use led to decreased post-operative pain. Upton et al. and Daccache et al. both had results that supported this idea. Specifically, Upton et al. observed that patients who underwent ANI-guided fentanyl administration for lumbar discectomies or laminectomies had significantly lower NRS scores, lower post-operative fentanyl administration, lower nausea scores, and lower incidence of shivering compared to the control group (standard practice) (33). In Daccache et al., 155 out of 180 patients that received intra-operative ANI-guided fentanyl for elective surgeries did not receive any post-operative opioids for pain; additionally, the cohort's maximal pain NRS score was 2 at 24 h post-surgery (34) (Tables 4, 5).

**Table 4:** Summary of post-operative pain with intra-operative ANI Use.

Study	Decrease in post-operative pain?	Post-operative time frame for pain scoring (hours)	Comparing post-operative pain scores with and without ANI use	Sample size
Upton et al. (33)	Yes	0–1.5	−1.3^a^ [−0.4 to 2.4]; *p* = 0.01	50
Daccache et al. (34)	Yes	Not specified	“At 24 h, the maximal NRS pain score was 2. One hundred and fifty-five patients (86%) did not receive any postoperative opioids.”	158
Gall et al. (2017) (24)	No	Not specified	0.00^b^ [−0.89 to 0.89]	60
Dostolova et al. (2019) (25)	No	Not specified	−0.04^b^ [−0.31 to 0.23]	72
Tribuddharat et al. (2021) (26)	No	0–1	“The respective NRS at the PACU among groups was similar.” *p* = 0.624	60
Szental et al. (2015) (27)	No	0–1	−1.4^c^ [−4.7 to 2.0]; *p* = 0.42	119

Brackets denote 95% confidence intervals [95% CI].

^a^ Denotes average change in NRS scores with ANI use compared to no ANI use.

^b^ Denotes mean difference in pain scores with ANI use compared to no ANI use, based on estimation.

^c^ Denotes mean difference in pain scores with ANI use compared to no ANI use obtained from study.

**Table 5:** Summary of post-operative opioid sparing with intra-operative ANI use.

Study	Decrease in post-operative opioids?	Post-operative hours studied	Opioid used	Sample size
Daccache et al. (34)	Yes	0–24	Oxycodone	180
Gall et al. (2017) (24)	No	0–24	Morphine	60
Dostalova et al. (25)	No	0–48	Morphine	72
Szental et al. (2015) (27)	No	0–1	Morphine/Tramadol/Ketamine	120

Other studies had contradictory findings. In Gall et al. and Dostalova et al., the studies previously discussed that found ANI-guidance decreased intra-operative opioid use, compared post-operative pain between patients with and without ANI-guided analgesia using the NRS scale and visual analog scale (VAS), respectively (24, 25). Despite both studies primary outcomes supporting less opioid use through ANI guidance, there was no significant difference in post-operative pain scores between the ANI groups and the control groups. Tribuddharat et al. and Szental et al., the studies discussed in the previous section that did not find a significant difference in intra-operative opioid use between ANI-treated and control groups, also did not see a significant difference in pain scores between ANI-treated and control groups (using NRS and VAS to assess pain, respectively) (26, 27) (Tables 4, 5).

### 3.3. Intra-operative ANI use and post-operative outcomes

There are a few studies that evaluated post-operative outcomes in patients that had ANI-guided analgesia. An observational study by Ramos-Luengo et al. found that patients whose ANI values were higher than 50 for at least 60% of the time under anesthesia had a significantly lower length of stay post-operatively (35). While this study didn't test whether an ANI-treated group had a lower length of stay compared to a control group, this study could suggest that ensuring ANI values stay above 50 for a significant amount of time intra-operatively can reduce a patient's length of stay. This isn't generalizable to all surgeries, however, as this was studied in patients undergoing varicose vein intervention. In Yang et al., elderly patients that had ANI guidance during spinal surgeries were found to have a higher post-operative neurocognition (36).

### 3.4. Intra-operative ANI use and post-operative outcomes in pediatric patients

In pediatric patients, some of the post-operative outcomes of ANI that were assessed include post-operative pain and agitation. As mentioned previously, Gall et al. found that children admitted in the PACU after surgery had significantly lower ANI scores (indicative of nociception) compared to children admitted in the PACU after medical imaging, where no painful stimulus occurred. Additionally, they observed a statistically significant negative linear relationship between ANI values and FLACC scores, further supporting ANI's ability to evaluate post-operative pain in pediatric children (30). Larsen et al. assessed whether ANI-guided analgesia would affect post-operative agitation based on the Richmond Agitation-Sedation scale in pediatric patients (37). ANI-guided analgesia led to a decrease in the number of children who had emergence agitation (9 out of 30 children) compared to the control group (15 out of 31 children). However, it is important to note that this was not statistically significant (*p* = 0.070). Furthermore, it is also important to note that the ANI-treated group of children had a statistically significantly higher average dose of fentanyl. However, this can also be interpreted as ANI-guidance allowed anesthesiologists to administer the most appropriate amount of opiates, which is reflected in the lower emergence agitation frequency.

## 4. ANI in the ICU and COVID-19

### 4.1. Validation of ANI to detect nociception in ICU patients

In the ICU setting, there are numerous studies that validates ANI ability to measure nociception (Table 6). Jendoubi et al. and Broucqsault-Dédrie et al. assessed ANI changes in response to painful stimuli in deeply sedated patients on mechanical ventilators and found that ANI significantly decreased (indicative of nociception) during painful stimuli compared to ANI values at rest (38, 39). Interestingly, Jendoubi et al. found a significantly negative correlation between ANI values and the behavioral pain scale (BPS) measurements (*r*^2^ = −0.469, *p* < 0.001), whereas Broucqsault-Dédrie et al. did not. However, it is worth noting that one major difference between the two studies is that the patients in Jendoubi et al. all had traumatic brain injuries, whereas the patients in Broucqsault-Dédrie et al.'s were from two different medical ICUs.

**Table 6:** Summary of ANI's validity in detecting nociception in adult ICU patients.

Study	ANI reflects nociception?	Noxious stimuli	Pain assessment	Sample size
Jendoubi et al. (38)	Yes	Mechanical Ventilation, Tracheal Suctioning	BPS	21
Broucqsault-Dédrie et al. (39)	Yes	Mechanical Ventilation, Patient Turning	BPS	41
Chanques et al. (40)	Yes	Dressing Changes, Patient Turning, Tracheal Suctioning	BPS	110
Papaioannuou et al. (41)	Yes	Burn Wound Treatment	NRS	20
Boselli et al. (2021) (42)	Yes	Closed-Tracheal Suctioning	N/A	15

In contrast to the previous studies examining deeply sedated patients on mechanical ventilators, Chanques et al. and Papaioannuou et al. validated ANI's effectiveness in detecting nociception in non-comatose, communicative ICU patients (40, 41). For Chanques et al. specifically, they found that “ANIi,” which they defined as the ANI average “calculated over a shorter period of time (64 s)” had a significantly negative correlation with the BPS scale (*r* = −0.30; *p* < 0.001) during routine care procedures (dressing change for a central venous/arterial catheter, turning a patient over, tracheal suctioning in intubated patients) (40). Interestingly, “ANIm,” defined as “an average calculated over the previous 4 min,” had no significant change during these procedures nor correlation to the BPS scale.

### 4.2. Application of ANI with COVID-19 ICU patients

In more recent times, ANI had application in COVID-19 studies as well (Table 6). Boselli et al. (2021) retrospectively analyzed how the ANI values would change during closed-tracheal suction of 15 ICU patients who had severe COVID-19 pneumonia (42). Through ANI, the authors found that ANI significantly decreased during tracheal suctioning, leading them to conclude that ANI can be a useful tool in detecting nociception during this procedure in COVID-19 ICU patients. In another study, Aragón-Benedí et al. assessed whether ANI monitoring could predict the outcomes of 14 critically ill patients with COVID-19 in the surgical ICU (43). These patients had their “ANIm” values (defined as “the mean ANI of the last 240 s”) measured for 240 s in a morning before their daily washing, and 30 days after this measurement the researchers examined whether each patient was still alive. From this, they categorized the patients into a “survivor” and “non-survivor” group. This study concluded that in critically ill COVID-19 patients, there was higher parasympathetic tone (as indicated by higher ANI values), potentially due to low sympathetic activity. In these patients with low sympathetic activity, there were higher mortality rates. However, it is important to note that this study has a very small sample size (*n* = 14), and thus this finding should not be generalized about COVID-19 related mortalities.

## 5. ANI application in maternity patients

There are a few studies that evaluated ANI's application in maternity patients as well. Le Guen et al. evaluated both the subjective VAS and the objective ANI values of patients in labor in four instances at 5 min intervals, regardless whether or not the patient was experiencing uterine contractions (44). This study found that the pain scores from the VAS model were significantly higher (signifying nociception) when patients experienced uterine contractions and that ANI values were significantly lower (signifying nociception) during uterine contractions. Furthermore, there was a statistically significant inverse correlation between the VAS and ANI values recorded, supporting ANI's application in this setting.

ANI was also applied in a study that assessed whether mother-infant skin-to-skin contact immediately after birth had an effect on maternal comfort (45). By using ANI, Vamour et al. found that the median ANI values at the end of skin-to-skin contact was significantly higher than before skin-to-skin contact, suggesting the importance of skin contact for mothers immediately after giving birth and further support for ANI's use in evaluating discomfort and pain.

## 6. ANI and septic patient outcomes

One potential application of ANI could be in predicting the outcomes of septic patients. In one study by Pontet et al. (2003), HRV was measured using an electrocardiogram in 47 septic patients starting from the beginning of their ICU admission (46). This study found that septic patients who developed multiple organ dysfunction syndrome (MODS) had a significantly decreased HRV at the time of ICU admission compared to patients who did not develop MODS. In another prospective study by Chen et al., 132 septic patients had their HRV measured using ECG shortly after admission into the emergency department and the study. In this study, patients who did not survive were found to have significantly lower HRV values compared to those that did survive (survival was defined as patients who were discharged in less than 28 days or remained alive for more than 28 days) (47). In another study, Annane et al. (1999) also found that septic shock patients had a significant reduction in HRV (48).

## 7. Discussion

### 7.1. ANI in the intra-operative setting

ANI has excellent potential in the intra-operative setting. In an artificial intra-operative setting, ANI is sensitive to the introduction of a noxious stimulus; however, the changes in ANI after an artificial stimulus cannot predict a significant hemodynamic response (5–8). It is possible that in these studies did not provide a stimulus strong enough to convert changes in sympathetic nervous system signals to a change in hemodynamic responses. In the future, studies will need to change the nature of the stimulus (such as duration, amplitude, and type) to see if ANI is context dependent.

In a real-life intra-operative setting, ANI was sensitive (i.e., decreased) during nociceptive stimuli during surgery in adult and pediatric patients (14–17). ANI's sensitivity to the presence of a noxious stimulus is not dependent on the method of intubation and returns to baseline by the end of the procedure (14, 21). However, the latency period between a change in ANI and a change in a patient's hemodynamic state is not known. Furthermore, the correlation between latency period and the amplitude of the hemodynamic change as not been studied. It is possible that longer latency periods could result in smaller amplitudes and could explain situations where a patient does not have a hemodynamic change after a nociceptive stimulus. Interestingly, the studies that utilized artificial stimuli found evidence that did not support ANI's ability to predict hemodynamic changes, whereas the adult studies in clinical settings did support ANI's predictive capabilities. It is also important to note that each study presented with various limitations: for instance, many of them mentioned a small sample size for their study. Among the studies mentioned, only the study by Ledowski et al. showed that the decreased ANI values from noxious stimulation is reversed by opioid administration (9). There is also no evidence that ANI changes are independent of patient fluid status. Overall, it appears that ANI may have clinical benefit in detecting nociception; however, hemodynamic predictive capabilities still appear controversial. As such, further studies should be conducted in a wider variety of clinical settings to further validate its use.

The effect of ANI on intra-operative opioid use is controversial. The use of different opioids (e.g., remifentanil, fentanyl, sufentanil), different surgical procedures that have varying length and nociceptive stimuli, and sample sizes could explain the different results (22–24). The results of studies that assess intra-opioid consumption using ANI could vary depending on the length of the surgery and the pharmacological properties of the different anesthetics used. Another important consideration is that while objective evaluation of intraoperative nociception may lead to changes in opioid dosing on individual patients, it may not result in an overall reduction in opioid dosing. Additionally, while no change in the average opioid dose may suggest that patients were dosed appropriately without ANI, ANI could allow more optimal titration of opioids for each patient, and potentially with better timing as well. It is also important to note that many studies assessed whether intra-operative ANI use can lead to decreased intra-operative opioid use; however, intra-operative opioid sparing could lead to undesirable outcomes such as increased post-operative pain and opioid use (49). As such, significant reduction in intra-operative opioid consumption may not be the most reflective endpoint (nor should it be the only endpoint) to assess ANI's ability to improve patient outcomes and presents a potential limitation to the applicability of these studies. After all, intra-operative opioid use is only one of many aspects in peri-operative pain management. Nevertheless, among the studies that utilized this endpoint to assess ANI's clinical relevance and found a significant decrease in opioid consumption, there was not a significant change in factors post-operation, such as side effects, complications, or recovery time (22–24). Other clinically relevant outcomes that could be assessed in future studies to improve our understanding in ANI's clinical relevance could be opioid-related adverse events and post-operative complications secondary to pain or sympathetic overactivation. However, given that these events are rare, many existing ANI studies are currently not powered for these outcomes.

### 7.2. ANI in the post-operative setting

ANI corelates well with subjective pain measures in the post-operative setting in both adult and pediatric patients (32–36). Furthermore, ANI-guided opioid administration can decrease post-operative opioid use (28, 29, 37, 38). However, there is contradictory evidence on whether intra-operative use of ANI-guided opioid administration decreases post-operative pain (measured by subjective pain scores) (28–31). In pediatric patients, one study also demonstrated a trend (although insignificant) between ANI-guided analgesia and lower post-operative agitation (41). The contradiction between decreased post-operative opioid use and unchanged subjective pain could be due to subjective nature of NRS and VAS. In the future, studies should replace subjective pain measures with more objective measures such as hemodynamic factors, ANI, and EEG signals.

Furthermore, there is a need to determine how ANI-guided opioid administration impacts patient outcomes. While Ramos-Luengo et al. found that ANI values could be related to longer hospital stays, the study's findings—that ensuring ANI values staying above 50 can reduce a patient's length of stay—are limited due lack of comparison to a control group and the fact that the study was conducted only on varicose vein interventions. For elderly patients that underwent ANI guidance during spinal surgery, it is possible that these patients have higher cognitive outcomes; however, the effect of ANI by itself is not clear (40). We believe that there needs to be significant more research to clearly understand the effect of ANI-guided anesthesia administrate on patient outcomes.

In pediatric patients, the current literature has varied findings on ANI's post-operative use and these applications of ANI should be studied more extensively before any conclusions could be made (34, 41). Additionally, it would be beneficial to explore the long-term effects of ANI-guided analgesia on post-operative outcomes in pediatric patients. The present narrative review mainly focused on short-term outcomes such as immediate post-operative pain and agitation. Investigating the impact of ANI on factors such as recovery time, length of hospital stay, and overall patient satisfaction could provide a more comprehensive understanding of the clinical benefits associated with ANI-guided analgesia in pediatric populations.

### 7.3. ANI in the ICU and COVID-19

The literature reviewed in this study provides evidence supporting the effectiveness of ANI in measuring nociception in the ICU setting. Studies on deeply sedated patients on mechanical ventilators demonstrated a significant decrease in ANI values during painful stimuli, indicating its ability to assess pain (42, 43). The correlation between ANI values and pain scales varied across studies, potentially influenced by patient population differences. ANI also showed promise in detecting nociception in non-comatose, communicative ICU patients (44, 45). In the context of COVID-19, ANI was found to be useful in detecting nociception during tracheal suctioning and showed potential in predicting outcomes in critically ill patients (17, 49). However, it is important to note that these COVID-19 studies had low sample sizes. Nevertheless, the current literature regarding ANI shows promising evidence for future application in research conducted in the ICU.

To enhance the utilization of ANI in the ICU, future research should focus on standardizing ANI measurement and interpretation, exploring correlations with pain scales across different patient populations and procedures, and conducting larger studies to validate its applicability, particularly in COVID-19 patients. In conclusion, ANI shows promise as a valuable tool for assessing nociception in the ICU setting. By addressing the identified areas for improvement, ANI can be optimized for pain management and improve outcomes in critically ill patients.

### 7.4. ANI application in under-studied patient populations

There are not many studies on the use of ANI in the maternity field. These studies have demonstrated ANI's potential in assessing pain and discomfort during labor and postpartum (14, 48). However, further studies should be conducted to evaluate ANI's validity in this unique patient setting given ANI's potential in this setting, such as evaluating how ANI changes because of epidural anesthesia or delivery, or if ANI could have some predictive power in maternity patient outcomes. In the future, standardization of ANI protocols and guidelines specific to this patient population would enhance its utility. Furthermore, ANI's relationship with other validated pain assessment tools in the maternity field should be explored to fully characterize its strengths and weaknesses in this patient population.

In septic patients, the studies consistently support the idea that a lower HRV in septic patients could lead to poorer outcomes (e.g., MODS or mortality). Given that ANI also measures HRV to assess the autonomic nervous system, there is potential for ANI use to have future application in the treatment of septic patients, and perhaps early evaluation of HRV through ANI in these patients could open opportunities to improve outcomes (15–17). Again, for a stronger conclusion of ANI's clinical use in septic patients, more studies are needed. In the future, larger-scale studies with diverse patient populations and ANI's correlation with other factors other than HRV are important to better understand ANI's place in sepsis management.

### 7.5. The existence of alternative available nociceptive monitoring technology

In addition to ANI, there exists numerous other novel alternative devices that propose to also monitor intraoperative nociception. These devices include, but are not limited to, the surgical plethysmographic index, nociception level index, the pupillary pain index, and the qNOX index (50–53). These devices offer both similar and different ways of interpreting nociception, particularly through modalities such as the plethysmograph amplitude, HRV, skin conductivity, pupillary diameter, and EEG physiology. Furthermore, they contrast from ANI in how they are set up as well; for instance, the surgical plethysmograph index utilizes only a pulse oximeter. Given the growing variety in which nociception can be assessed, it is important to note that ANI is not the sole device on the market that claims this purpose, and that each and every pain monitor should be well analyzed in their own studies and in different clinical settings to assess for their individual validities, applicability, and limitations in clinical medicine.

## 8. Conclusion

The literature regarding ANI's validity in reflecting nociception is mostly supportive both intra-operatively and in other clinical settings. In terms of whether intra-operative ANI use makes a significant difference in intra-operative opioid consumption, the literature is mixed in its clinical benefits. For post-operative pain and opioid use, many studies have not found a clinical benefit using ANI intra-operatively. Additionally, applications of ANI have also been found to be useful in other settings and patient populations, such as in COVID-19, maternal, and septic patients; however, it is important to note that these studies are limited in number and cannot be generalized.

The potential for the various novel nociceptive monitoring devices to improve patient care is an exciting advancement in the field of surgery and anesthesia. Many studies were conducted on the validity and applications of those alternative devices as well; in this narrative review, we focused primarily on a critical analysis of the ANI device. Despite all the findings presented in this review, each study presents with their own limitations, such as different patient populations, clinical settings, nociceptive stimuli, opioid choices, and small sample sizes that makes generalizing ANI's clinical benefits difficult. There appears to be support for ANI's ability to reflect nociception, but further research on ANI's benefits and applications are necessary before larger conclusions can be made.
